# The American and Colonial Journals: Surgery

**Published:** 1840-01

**Authors:** 


### II. THE AMERICAN AND COLONIAL JOURNALS.

### SURGERY.

*Report of Cases of Un-united Fracture, treated at the New York Hospital.* By John S. Heard, m.d., late House-Surgeon.

The chief object of the following report is to offer to the profession an account of a new mode of treating un-united fractures of the extremities, first practised in the New-York Hospital, and, so far as my knowledge extends, still confined to the practice of that institution.

[The author, after stating the general causes of non-union, and the objects of treatment, passes in review the different methods heretofore employed: viz., blisters; friction of the extremities of the bones; rest combined with pressure; salivation; seton; introduction of a silver wire between the fragments; injection of stimulant liquids; application of caustics; sawing off the extremities.]

All these different methods having occasionally failed in effecting bony union,—it occurred to Dr. J. Kearny Rodgers, one of the surgeons of the New-York Hospital, that if, after removing the extremities of the fragments and exciting their periosteum, he could keep them in apposition for a certain period,—firm osseous union would ensue. He accordingly, after sawing off the extremities of the fragments, drilled a hole in the end of each, and passing a silver wire through, drew the bones together, and kept them in co-aptation until firm union was effected. Subsequently, Drs. Mott and Cheesman, colleagues of Dr. Rodgers, were likewise successful in inducing bony union by the same operation. This operation has not, we believe, been adopted in any other place than the New-York Hospital. Indeed, the only notice of it that I have been able to meet with, is in a report of a clinical lecture by Mr. Liston, in which he makes these remarks: " A proposal has, I think, been made, to denude the ends of the bones, drill holes in them, and connect them by a silver wire. It is too artificial a mode of proceeding, and I should fear, that from exposure and bruising of the ends necessary for accomplishing the object, (for perforation cannot be made unless by denuding the bone freely, and holding it securely by some contrivance until the object is accomplished,) that necrosis, instead of union, would be apt to follow." Now, what Mr. Liston means by " too artificial a mode of proceeding" is not very apparent: in what is it more artificial than Mr. White's method? In the simple passing of a silver wire: and is an operation, although successful, to be abandoned, because it is artificial? Surely this rule would have a very limited application in surgery. And as regards "necrosis instead of union" being more likely to follow, it will be sufficient to state, that this operation has hitherto never failed of success. And are we to refuse to adopt a sure plan of treatment, although it be severe, for one which, though less so, still, occasionally, is of no avail?

[The paper is illustrated by the narration of several cases. We can only afford room for the first two.]

Case I.—George Westerfield, aged 15, admitted into the New-York Hospital, under Dr. Rodgers, July 25, with an un-united fracture of the right os brachii, about two inches above the elbow-joint. This accident was the result of a limb of a tree falling upon his arm in Ohio, in December, 1824. From his account, it appears that a medical man reduced the fracture immediately, and placed splints on the arm, which was borne in a sling. At the expiration of twelve days, the dressings were removed, and the union, which he seems to have expected, not having taken place, the ends of the fractured bone were rubbed together, and the splints re-applied. This treatment was persevered in several months. In June, 1825, a seton was passed between the ends of the bone, and withdrawn in a week; splints were worn for four weeks, but no union ensued. He remained without treatment from this time until his arrival in New-York, in June, 1826; in the latter end of June, a seton was passed by a surgeon of this city, and retained for six months, motion being prevented by splints applied to the arm. The seton having been fairly tried, and removed without any beneficial result, the patient almost despaired of ever recovering the use of his arm, but was willing to submit to any expedient which might be proposed likely to bring about union. It appeared to Dr. Rodgers, that but one other mode offered any prospect of success, and that was Mr. White's plan of sawing off the ends of the bone, and reducing the parts to the state of a recent compound fracture. On the 31st of July, Dr. R. made an incision three inches in length, through the integuments of the arm, on the outer edge of the biceps muscle, down to the bone. The lower end was easily turned out of the wound, and half an inch sawed off. The upper end could not be sawed off in the same way; but having guarded the soft parts by a thin slip of wood passed down on each side of the bone, about half an inch was removed by a circular saw. It being found impossible to bring the ends of the bone in contact, in consequence of a slip of muscle passing between them, this was divided; but the fragments, although placed in apposition, soon regained their former bearings an inch and a half asunder. Apprehensive that he should be foiled if the fragments were allowed to remain thus far apart, Dr. R. drilled a hole into the medullary cavity through the shell of each end; through these holes a silver wire was passed, and the ends of the bone retained in co-aptation. The ends of the wire were drawn through a canula, which remained in the wound. The os brachii was much softer than natural, and it was feared that this state would prevent any ossific deposit. The wound was dressed with adhesive straps; and the arm, enveloped in splints, was placed on a right-angled splint properly hollowed out.

August 15th. The canula fell from the wound with the loop entire, so that the bone must have broken away; the bones however continued in proper position; not expecting bony union for some time, the arm was not examined for a month; during this time the wound had almost healed.

October 8th. Sixty-nine days after the operation, Dr. Rodgers was gratified to find that the bone had united, but he was unwilling to test its strength by force.

October 16th. On examination to-day the limb was found to have united perfectly. The patient was not allowed to move from his bed for two months after the operation.

December 3d. The patient was presented to the clinical class, with every motion of the arm perfectly regained, the os brachii being two inches shorter than its fellow.

CASE II.—Charles Grill, born in Germany, aged 26, a cutler, was admitted into the New-York Hospital, September 28th, 1838, with a simple fracture of the radius of the right forearm, about two inches above the wrist joint. The injury was caused by his hand being caught in machinery and bent forcibly back. There was considerable contusion of the whole forearm, and some laceration of the hand. The fracture of the radius was very oblique, and the lower end of the upper fragment projected nearly through the integuments. The fracture was reduced, and the patient being placed in bed, the arm was laid in a flexed position on a pillow, covered with oil silk, and lot. evap. applied. As soon as the state of the arm would allow, the requisite pads and splints were applied, and the arm supported in a sling. At the expiration of four weeks from the application of the splints, they were removed, but no union had taken place. It was now suggested that a strip of muscle interposed between the fragments was the cause of non-union. A splint, with a hand-piece setting off from the body of the splint at an angle of about 110°, was now applied upon the palmar side of the forearm, in order to elevate the lower fragment. The interosseous pads being only applied between the ulna and this lower fragment, the usual splint was placed on the dorsal side. The arm was thus supported in a sling for six weeks; at the expiration of which period, upon removing the apparatus, there was still no union. The same apparatus was replaced, and the patient put under the use of mercury, but it produced profuse diuresis, and was discontinued. The splints were allowed to remain applied until January 10th, when Dr. Rodgers made an incision about two and a half inches in length, over the seat of fracture on the palmar side of the forearm; upon exposing the fragments, the radial artery was found running between them; the lower fragment was drawn down to the ulna, while the upper one was separated near an inch from it, a slip of the extensor carpi radialis muscle intervening. The radial artery was divided and secured at both extremities, the intervening muscle was severed, and the ends of the bone removed with the bone pliers. A hole was now drilled into each extremity, and a silver wire being passed through, the bones were approximated by twisting the wire, the end of which was allowed to hang from the wound. The edges of the wound being brought together by adhesive straps, the ordinary pads and splints were applied, and the arm suspended in a sling. A slight attack of erysipelas occurring, required the confinement of the patient to bed and the removal of the splints; upon the subsidence of the erysipelas in a few days, the splints were re-applied.

February 2d. The wire came away to-day the loop being entire. The splints and bandages were continued until April 2d, when, upon removing them, the union was found to be perfect. He remained in the hospital, using passive motion, until April 15th. *New York Journal of Med. and Sur. Oct.* 1839.

